# Development of a Colloidal Gold-Based Immunochromatographic Strip for Rapid Detection of H7N9 Influenza Viruses

**DOI:** 10.3389/fmicb.2018.02069

**Published:** 2018-08-31

**Authors:** Zhihao Sun, Baolan Shi, Feifei Meng, Ruonan Ma, Qingyun Hu, Tao Qin, Sujuan Chen, Daxin Peng, Xiufan Liu

**Affiliations:** ^1^College of Veterinary Medicine, Yangzhou University, Yangzhou, China; ^2^Jiangsu Co-innovation Center for the Prevention and Control of Important Animal Infectious Disease and Zoonoses, Yangzhou, China; ^3^Jiangsu Research Centre of Engineering and Technology for Prevention and Control of Poultry Disease, Yangzhou, China; ^4^Sinopharm Yangzhou Vac Biological Engineering Co., Ltd., Yangzhou, China; ^5^Joint Laboratory Safety of International Cooperation of Agriculture and Agricultural-Products, Yangzhou, China; ^6^College of Veterinary Medicine, Cagayan State University, Tuguegarao, Philippines

**Keywords:** H7N9, influenza, monoclonal antibody, epitope, gold immunochromatographic strip

## Abstract

Both high- and low-pathogenic H7N9 influenza A virus (IAV) infections have been found in human and poultry in China, and most human cases are related to contact with infected poultry. It is necessary to develop a rapid and simple method to detect H7N9 IAV in poultry. In this study, 13 monoclonal antibodies (McAbs) against the H7N9 IAV hemagglutinin were developed, and three critical amino acid epitopes (198, 227, 235) were identified based on the reactivity of these variant and wild-type strains with the McAbs. We developed an immunochromatographic assay for H7N9 AIVs using two McAbs recognizing the epitope position 227 and 235. The assay had good specificity, stability, and sensitivity, with a detection limit of swab and tissue samples of 2.5 log_10_EID_50_/0.1 mL, which is suitable for the analysis of clinical samples. This assay provides an effective method for the rapid detection of H7N9 AIVs in poultry.

## Introduction

The H7 subtype of the influenza A virus (IAV) HA gene has been found in combination with all nine NA subtype genes and all H7Nx combinations have been found in wild birds, the natural reservoir of the virus (Abdelwhab et al., 2014). Live-poultry markets (LPMs) bring together numerous bird and poultry species from different sources in a high-density setting, thus providing an ideal environment for reassortment among avian influenza viruses of different subtypes (Shi et al., 2013). The novel influenza A H7N9 viruses first reported in March of 2013 have led to five human epidemics (Wang et al., 2017). There is some evidence that the H7N9 AIVs can be directly transmitted from poultry to humans and cause disease although they are of low virulence to poultry (Kurtz et al., 1996; Banks et al., 1998; Chen et al., 2013). An H7N9 virus with a four basic amino acid insertion in a host protease cleavage site in the hemagglutinin protein was identified in two patients with H7N9 virus infection on February 19, 2017 (Zhang et al., 2017). Animal infection experiments confirmed that such H7N9 viruses have high virulence in chickens and variable virulence in mice, suggesting a new potential threat to public health and the poultry industry (Liu D. et al., 2018; Liu J. et al., 2018).

Molecular and serological methods have been developed for detecting H7N9 AIVs (Graaf et al., 2017; Jia et al., 2017; Liu J. et al., 2018). Molecular tests include conventional reverse transcriptase polymerase chain reaction (RT-PCR) and real-time RT-PCR (rRT-PCR). For laboratory diagnosis and surveillance, serological tests including the hemagglutinin inhibition (HI) test and enzyme-linked immunosorbent assay (ELISA) are also used (Munch et al., 2001; Shien et al., 2008; Velumani et al., 2008). Moreover, the HI test is also the gold standard suggested by the OIE^1^. However, these conventional methods are time-consuming, require specialized materials and equipment, and occasionally produce false-positive results. The gold immunochromatographic assay (GICA), a technique based on the specific antigen–antibody immunoreactions, is a highly useful tool in diagnostics that can be completed within 30 min, without specialized equipment or complicated handling procedures, providing convenience for rapid testing.

In this study, an inactivated H7N9 virus was used as the immunogen to prepare monoclonal antibodies (McAbs). Antigen epitopes of the McAbs were determined by using antigen escape tests and HA gene sequencing. McAbs with different epitopes were used to develop a gold immunochromatographic strip for detecting H7N9 AIVs.

## Materials and Methods

### Ethical Approval

The Jiangsu Administrative Committee for Laboratory Animals approved all animal studies (Permit Number: SYXKSU-2007-0005) according to the guidelines of Jiangsu Laboratory Animal Welfare and Ethical of Jiangsu Administrative Committee of Laboratory Animals.

### Viruses

The H7N9 LPAIV strain A/Chicken/Jiangsu/W1-8/2015 (CK/W1-8/15, accession number for HA gene: MG739458) was isolated from chicken in LPMs in 2015. Other subtype AIVs (**Supplementary Table S1**) and avian viruses such as Newcastle disease virus (NDV), adenovirus, avian infectious bronchitis virus (IBV), Marek’s disease virus (MDV), and avian infectious bursal disease virus (IBDV) were obtained from the Key Laboratory for Animal Infectious Diseases, Ministry of Agriculture, Yangzhou University, Yangzhou, China and used for specificity tests. All live highly pathogenic avian influenza viruses were handled in the authorized animal biosafety level 3 facilities at Yangzhou University.

### Monoclonal Antibodies

Monoclonal antibodies against H7N9 AIV were developed following a standard procedure (Harlow and Lane, 1988). Briefly, 6-weeks-old BALB/c mice were subcutaneously primed with 20 μg of inactivated H7N9 virus in Freund’s adjuvant twice with a 3-week interval, and splenocytes were fused with Sp2/0 myeloma cells 3 days after the last boosting of inactivated H7N9 virus without adjuvant. The hybridomas were screened and selected by hemagglutinin inhibition (HI) assay, and HI titers ≥ 4 were considered positive (Influenza, 2012). The ascitic fluids of positive hybridomas were generated in mice.

### Virus Neutralization Test

To identify neutralizing McAbs, the McAbs that yielded positive results in the HI assay were tested in virus neutralization (VN) assays (Throsby et al., 2008). Briefly, 10-fold serial dilutions of McAbs were mixed with an equal volume of 100 EID_50_ of CK/W1-8/15 virus allantoic fluid and incubated for 1 h at room temperature. The mixture was then injected into 10-days-old specific pathogen-free (SPF) embryonated chicken eggs and incubated for 72 h at 35°C. Allantoic fluids were tested for hemagglutinating activity, and HA titers ≥ 4 were considered positive. The neutralization titers were calculated according to Reed and Muench (1938), and VN titers < 10 were considered negative.

### Selection of Escape Mutants and Nucleotide Sequencing

Escape mutants were selected by incubating the McAbs with their parent virus CK/W1-8/15, essentially following the procedure of a previous study (Tsuchiya et al., 2001; Nakajima et al., 2007). Briefly, the diluent of parental virus (10^6^ EID_50_/0.1 mL) was mixed with an equal volume of ascites fluid containing McAbs. After incubation for 1 h at room temperature, the virus–antibody mixture was injected into 10-days-old SPF embryonated chicken eggs and incubated for 72 h at 35°C. Serial 10-fold dilutions of the positive allantoic fluid (10^-5^, 10^-6^, 10^-7^, and 10^-8^) were mixed with an equal volume of ascites fluid containing McAbs, then the above steps were repeated once. The antigenic character of each isolate was examined using the HI test. The HA gene sequence of each mutant was determined by PCR amplification and sequence analysis (Hoffmann et al., 2001), and the deduced amino acid sequence was compared with that of CK/W1-8/15 to identify the epitope recognized by the selective McAb. The escape mutants with more than one mutation in the HA gene were further confirmed by constructing point-mutated rescue viruses using reverse genetics (Hoffmann et al., 2000).

### Preparation of Colloidal Gold and Colloidal Gold Conjugation

Colloidal gold was obtained according to a method described previously (Paek et al., 2000). Briefly, the aqueous solution of chloroauric acid [0.01%, (wt/vol); 100 mL HAuCl_4_] was heated to the boiling point, followed by the rapid addition of 14 mL of 1% trisodiumcitrate solution with rapid stirring. The reaction mixture was boiled for another 10 min and gradually boiled until the color turned red. The colloidal gold solution was cooled to room temperature and then stored in a dark bottle at 4°C.

McAbs 1B6, 1A11, or 1A2 were add separately to 10 mL of colloidal gold solution and stirred for 30 min. An aqueous solution containing bovine serum albumin (BSA; 10%, wt/vol; 2 mL) was added to block endogenous colloidal gold reactivity. The mixture was then centrifuged at 12,000 rpm and 4°C for 15 min to remove any unbound antibody. The pellet was resuspended in 1 mL of 0.01 M Tris-HCl (pH 8.0; Ju et al., 2010).

### Preparation of the Immunochromatographic Strip

**FIGURE 1:**
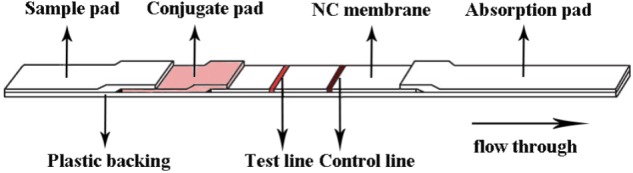
Schematic diagram of the immunochromatographic strip.

An immunochromatographic strip that includes a sample pad, a conjugate pad, a nitrocellulose membrane, and an absorbent pad was prepared as shown in **Figure 1**. First, the sample pad was saturated with a PBS solution (pH 8.5) containing Tween 20, 1% (wt/vol) BSA, and conjugated antibodies. The sample pad was then dried at 37°C for 1 h. Paired McAbs were microsprayed onto the NC membrane at a concentration of 0.8 mg/mL and localized to specific positions on the strip that were designated as the capture test line (**Figure 1**). An anti-IgG antibody was also microsprayed onto the same membrane at a concentration of 1.2 mg/mL and localized to the capture control line. The membrane was dried at 37°C for 2 h. Pure cellulose fiber was used as an absorbent pad. Immunochromatographic strips were store in a desiccator at 4°C prior to use.

### Specificity, Sensitivity, and Stability Evaluations of the Developed Strip

To evaluate the specificity of the strip, different HA subtypes of AIVs (H1, H3, H4, H5, H6, H8, H9, H10, H11, and H12), serial H7N9 AIVs isolated from year 2013 to 2017 and other avian viruses such as NDV, IBV, MDV, and IBDV were simultaneously tested. An aliquot of 100 μL of each sample was added to the strips and incubated for 15 min at room temperature.

Strip sensitivity was determined using a serial diluted of a positive H7N9 AIV allantoic fluid sample (CK/W1-8/15, HA titer = 256, 10^5^ TCID_50_). The allantoic fluid sample was diluted 1:2, 1:4, 1:8, 1:16, 1:32, 1:64, 1:128, 1:256, 1:512, and 1:1024 with 0.01 M PBS, respectively. These strips were tested to determine their sensitivity in detecting allantoic fluid samples of H7N9 subtype AIVs upon storage at room temperature for 12 months.

### Detecting Swab and Lung Samples From Experimentally Infected Chickens

Three-weeks-old SPF White Leghorn chickens purchased from Beijing Meiliyaweitong Experimental Animal Technology Co., Ltd, were inoculated intranasally with 10^6^ EID_50_ of AIV H7N9 in a 0.2 mL volume (*n* = 10). Trachea and cloacal swabs were collected from chickens at 1, 3, 5, and 7 days post-infection (dpi) and resuspended in 1 mL PBS. In addition, three other chickens were euthanized at 1, 3, 5, and 7 dpi, and the lung samples of infected chickens were subsequently harvested and homogenized in 1 mL PBS. The viral titers of swab and lung samples were determined by EID_50_. An aliquot of 100 μL of each sample was also added to the strip and incubated for 15 min at room temperature.

### Evaluation Using Clinical Swab Samples

Cloacal swabs (*n* = 200) were collected from apparently healthy poultry in a LPM of Jiangsu province. The swabs were collected in 1 mL PBS supplemented with antibiotics (penicillin 10,000 unit/mL, streptomycin 10 mg/mL, gentamycin 250 μg/mL, kanamycin 250 μg/mL) and subjected to strip detection and virus isolation. After virus isolation, the subtypes of AIV isolates were determined by HI assay or HA gene sequencing.

## Results

### Selection and Characterization of Monoclonal Antibodies

Thirteen McAbs against CK/W1-8/15 HA were generated. The HI titers of the McAbs ranged from 6–13 log2 and the VN titers of the McAbs ranged from 20–2000, indicating that the McAbs were capable of inhibiting the wild type virus in both HI and VN assays (**Table 1**).

**Table 1:** Biological properties of H7-specific McAbs generated in this study.

McAb^a^	Isotype	HI titer (log2)	VN titer
1C8	IgG_2a_	13	100
1B6	IgG_2a_	13	316
1A2	IgG_2a_	12	100
1C3	IgG_2b_	10	50
1B9	IgG_2b_	8	200
1C9	IgG_2b_	8	25
1D12	IgG_2b_	9	200
1A11	IgG_1_	6	20
1H11	IgG_1_	7	200
2F8	IgG_1_	7	64
1B10	IgG_2a_	10	1250
1F12	IgG_2a_	11	2000
3G4	IgG_2a_	10	640

*^a^Untreated mouse ascetic fluid of each hybridoma was used in HI and VN assays. Titers shown were the reciprocals of the highest dilutions showing HI or VN activity against CK/W1-8/15 (H7N9) virus.*

### Escape Mutants

Escape mutants were selected by inoculating embryonated SPF chicken eggs with CK/W1-8/15 in the presence of the McAbs. HI titers of the McAbs to these resulting mutants were significantly reduced or abolished (**Table 2**). The result of HA gene sequencing of mutants showed that 13 mutants possessed either one or two amino acid mutations. After confirmation by point-mutated rescue viruses, the positions 235L, 227G, or 198A were found to be the critical amino acid for McAbs recognition.

**Table 2:** Amino acid mutations in the HA of escape mutants from CK/W1-8/15 (H7N9).

Mutants	HI titer (log2)^a^	Mutations
m1C8	1	G214E^b^,L235Q
m1B6	1	G214E,L235Q
m1A2	2	S136G,G227E
m1C3	0	A169T, L235Q
m1B9	0	A169T, L235Q
m1C9	0	A169T, L235Q
m1D12	1	A143T, L235Q
m1A11	2	A198E
m1H11	0	A198E
m2F8	1	A198E
m1B10	0	L235Q
m1F12	2	L235Q
m3G4	0	L235Q

*^a^The HI titers obtained with each selecting McAb against mutants.*

*^b^H7 numbering.*

### Specificity, Sensitivity, and Stability Evaluations of the Developed Strip

**FIGURE 2:**
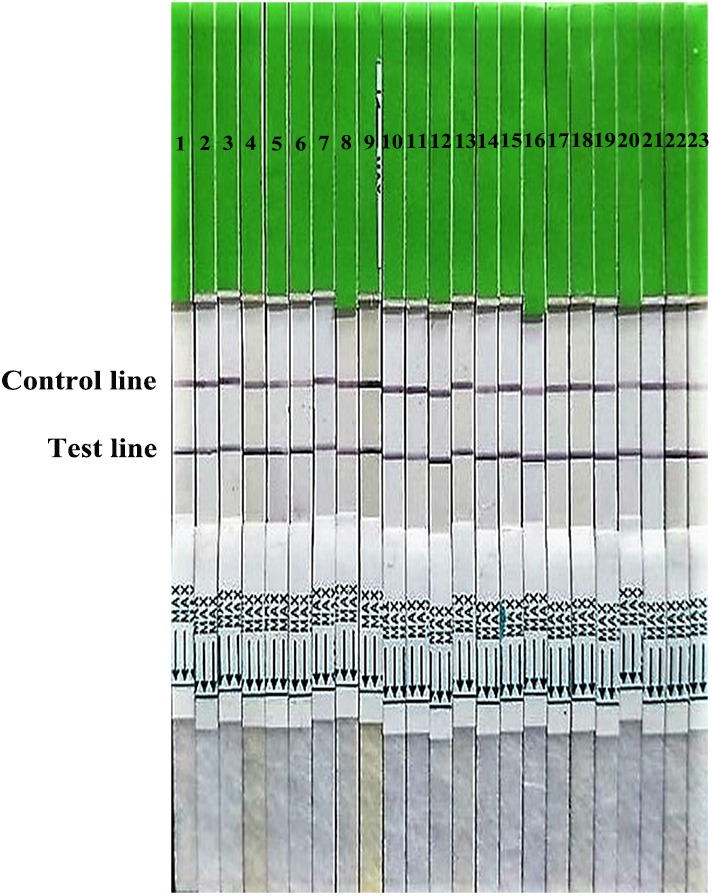
Broad reaction of the strip for H7 subtype AIVs isolated from 2013 to 2017. 1–9: isolated in 2013; 10–17: isolated in 2014; 18, 19: isolated in 2015; 20, 21: isolated in 2016; 22: isolated in 2017; 23: A H7N9 HPAIV stain A/chicken/Hebei/XT-3/2017 (CK/XT-3/2017).

**FIGURE 3:**
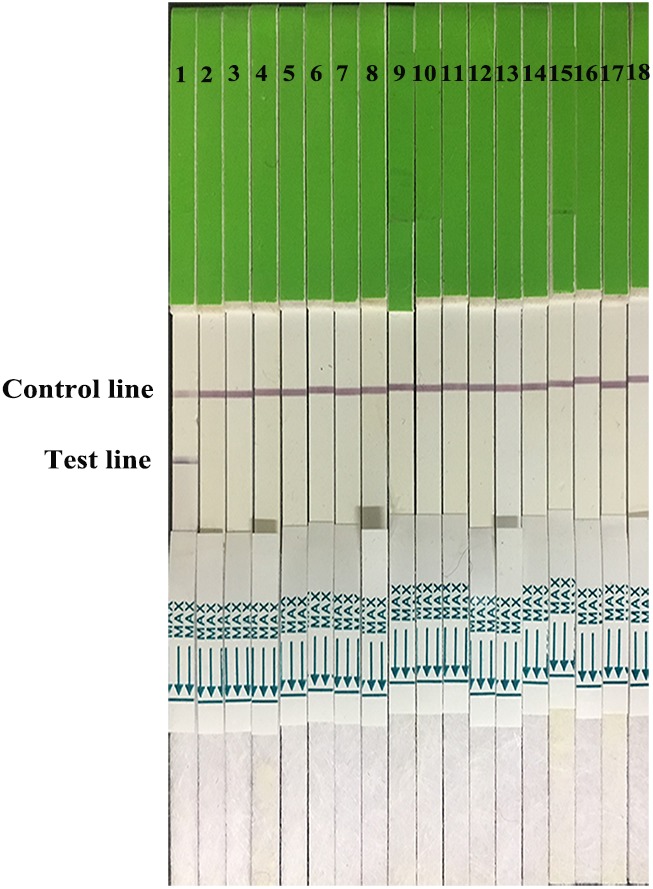
Strip specificity. 1: H7 positive AIV. 2–12: H1 AIV, H3 AIV, H4 AIV, H5 AIV, H6 AIV, H8 AIV, H9 AIV, H10 AIV, H11 AIV, and H12 AIV. 13–16: NDV, IBV, MDV, and IBDV. 17, 18: PBS and water.

Three McAbs 1B6, 1A2, 1A11, which recognized positions 235L, 227G, or 198A, respectively, were screened for conjugation or capture antibodies. After optimization, the McAbs 1B6 and 1A2 were selected as conjugation and capture antibodies, respectively. The specificity results revealed that only H7N9 subtype AIV isolates showed two red lines in the test and control area, while other subtypes AIV and other non-AIV strains showed a single red line in control area (**Figures 2**, **3**), indicating that the gold immunochromatographic strip had high specificity for detecting H7N9 AIVs.

**FIGURE 4:**
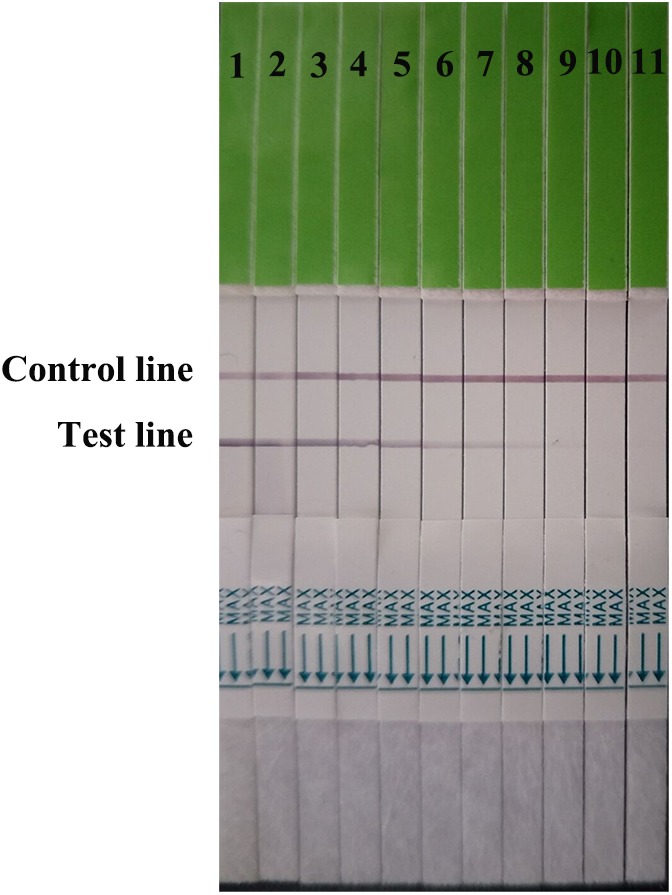
Strip sensitivity. Serial dilutions of the positive sample ranging from 2^1^ to 2^10^ were used to determine the sensitivity of the strip. The titer of the positive sample was 2^8^. 1: the positive sample. 2–11: diluted ranging from 2^1^ to 2^10^.

**FIGURE 5:**
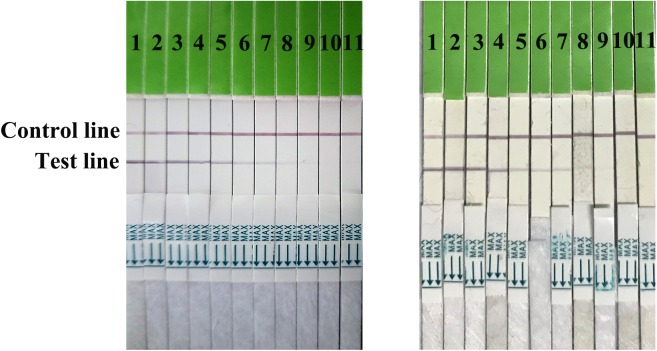
Strip stability. The sensitivities of fresh strips (left) and strips after 12 months of storage (right) were determined. 1: the positive sample. 2–11: diluted ranging from 2^1^ to 2^10^.

Serial dilutions of the positive H7N9 AIV sample ranging from 2^1^ to 2^10^ were used to determine the sensitivity of the strip. Compared to the hemagglutination test, a HA unit or 10^2.6^ TCID_50_ of H7N9 AIV (1:256 dilution) was successfully detected by using the strip (**Figure 4**), indicating that the gold immunochromatographic strip had high sensitivity. After 12 months of storage, the strips still had the same detection limit for H7N9 AIVs as freshly produced strips (**Figure 5**).

### Detection of Swab and Lung Samples From Infected Chickens

**FIGURE 6:**
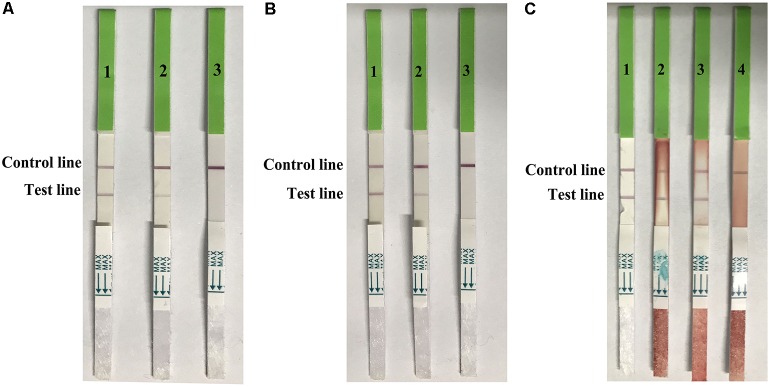
Use of the strip for detecting trachea swabs **(A)**, cloacal swabes **(B)**, and lung samples **(C)** from H7N9 AIV experimentally infected chickens. A-1, B-1, and C-1: H7 positive AIV, A-2: trachea swab sample contained 2.5 log_10_ EID_50_, A-3: trachea swab sample contained 1.5 log_10_EID_50_, B-2: cloacal swab sample contained 2.5 log_10_EID_50_, B-3: trachea swab sample contained 1.5 log_10_EID_50_, C-2: lung sample contained 3.5 log_10_EID_50_, C-3: lung sample contained 2.5 log_10_EID_50_, and C-4: lung sample contained 1.5 log_10_EID_50._

The distribution of viruses in the swabs (trachea and cloacal) and lung samples were detected at 1, 3, 5, and 7 dpi (**Table 3**). Virus titers ranged from 1–2.5 log_10_EID_50_/0.1 mL in the swabs, and 1.5–3.5 log_10_EID_50_/0.1 mL in the lung samples. The detection limits in both swabs and lung samples in the strip test were 2.5 log_10_EID_50_/0.1 mL (**Figure 6**). These data suggested that the developed strip was suitable for detecting H7N9 AIVs from infected samples.

**Table 3:** Virus titers in swab and lung samples from infected chickens.

Virus strain	Virus titer (log10 EID_50_ of virus ± SD/0.1 mL)											
	1 dpi^a^			3 dpi			5 dpi			7 dpi		
	Trachea	Cloacal	Lung	Trachea	Cloacal	Lung	Trachea	Cloacal	Lung	Trachea	Cloacal	Lung
W1-8	5/10^b^ (1.6 ± 0.7)	0/10	1/3(2.5)	6/10 (1.8 ± 0.7)	2/10 (2 ± 0.5)	3/3(3.2 ± 0.5)	6/10 (1.4 ± 0.2)	1/10 (1.5)	3/3(2.5)	3/10 (1.3 ± 0.2)	1/10 (1.5)	1/3(1.5)

*^a^dpi = days post-infection; ^b^positive number/tested numbers.*

### Detection of Clinical Samples From LPM

A total of 200 cloacal swab samples were detected by using the strip and the HA subtypes of AIVs were confirmed by isolation and sequencing (**Table 4**). The results of the strip assay showed that 3% (6/200) of poultry samples from the LPM were positive for H7N9 AIV, consistent with 4.5% (9/200) as determined by virus isolation, and 3.5% (7/200) as determined by sequencing. Therefore, the sensitivity of the strip compared to the HI assay and PCR assay was 66.7 and 85.7%, and the specificity was 98.5 and 99.5%, respectively. Of the six positive samples determined by the strip assay, two samples contained H7N9 AIVs only, while the other four samples contained H7N9 and H5 subtype AIVs.

**Table 4:** Results from cloacal swab samples from LPM using the strip assay, isolation, and gene sequencing.

Isolates	Host	The strip assay	HA subtype	Gene sequencing
1	Chicken	+^a^	H7	H7
2	Chicken	+	H5/H7	H5/H7
3	Chicken	-^b^	H5/H7/H9	H5/H7
4	Chicken	+	H5/H7	H5/H7
5	Chicken	+	H5/H7	H7
6	Chicken	-	H5/H7	H5
7	Chicken	-	H5/H7	H5
8	Chicken	+	H7	H7
9	Chicken	+	H5/H7	H5/H7

*^a^positive; ^b^negative.*

## Discussion

In 2013, the Chinese Food and Drug Administration (CFDA) approved three PCR kits for specific detection of H7N9 virus. However, no rapid immunoassays for clinical samples have been approved.

In this study, we developed an immunochromatographic strip based on the lateral flow platform using two specific H7-directed McAbs for antigen capture and detection. To avoid possible cross-reactivity and low affinity, the McAbs that recognized different surface epitopes were chosen by further analysis of escape mutants. A total of 13 McAbs were developed, and three epitopes 198A, 227G, and 235L were identified (**Table 2**). Even though the key amino acids of the epitopes were identical, the HI titers of the different McAbs against the same epitope of corresponding mutants were not the same, which may be due to epitope recognition issues.

Liu et al. (2015) mapped the five known epitopes of human H3N2 HA onto H7 HA based on the structural comparison of HAs and inferred 130 antigenic sites for the H7 subtype: 18, 22, 27, 41, and 22 antigenic sites for epitopes A, B, C, D, and E, respectively. Furthermore, mutations in epitope D had relatively low frequencies (Matrosovich et al., 1997). The positions of the McAbs we developed belonged to two epitopes (198 in epitope B, 227 and 235 in epitope D). We also quantified the spontaneous mutation levels for each of the three amino acid positions (**Table 5**) by comparing 642 full-length H7 sequences deposited in GenBank. The alignment results revealed that the position 227 had 0% variation rate and was conserved, compared to the other positions. We selected two McAbs 1B6 and 1A2 as conjugation and capture antibodies for the strip.

**Table 5:** Natural mutations at three key amino acid positions in the HA of H7N9 viruses.

Amino acid position^a^	Mutations (% of isolates bearing each residue)^b^
227	G (100), E (0)
235	L (86.14), Q (13.86)
198	A (92.06), T (7.94), E (0)

*^a^H7 numbering.*

*^b^A total of 642 full-length H7 sequences available in Genbank was analyzed.*

Kang et al. (2014) developed a rapid diagnostic test for the novel avian influenza A H7N9 virus in patients, with a limit of detection of 10^3.5^ pfu/mL or 10^3^ TCID_50_ of H7N9 virus culture supernatants in 15 min, less sensitive than our strips (10^2.6^ TCID_50_). Manzoor et al. (2008) developed a Pen-site Test Kit for the rapid diagnosis of H7 highly pathogenic avian influenza with a limit of detection of 4.5 log_10_EID_50_ for detecting both swab samples and tissue homogenates, less sensitive than our strips (2.5 log_10_EID_50_). Jin et al. (2014) also developed a GICA for H7N9 AIVs from infected patients, with relatively low sensitivity (33.3%) compared with RT-PCR, and not as good as the sensitivity of our assay (85.7%). Although the rapid immunoassay was less sensitive than rRT-PCR or virus isolation, the strips could detect the clinical samples from LPMs, which could be used as an indicator for H7N9 subtype AIV infections. Highly pathogenic H7N9 subtype avian influenza viruses spread in Southern China and led to massive death of domestic poultry, which may increase the risks for human infections (Quan et al., 2018). The strips could also detect the high pathogenic H7N9 subtype AIV (CK/XT-3/2017) from samples. All H7N9 subtype AIVs isolated from 2013 to 2017 were successfully detected by the strip, indicating a broad spectrum for H7N9 AIVs (**Figure 2**). Moreover, the strips did not detect the other HA subtype AIVs regardless of high or low HA titer (**Supplementary Table S1**). Therefore, the strip is suitable for detecting clinical samples of H7 subtype AIVs (including H7N9 AIVs), either from LPMs or from diseased chickens.

## Author Contributions

DP, TQ, SC, ZS, and XL conceived and designed the experiments. ZS, TQ, and FM collected samples. ZS, BS, FM, RM, and QH did the experiments. ZS, FM, and SC prepared the tables and figures. DP, ZS, TQ, and XL performed the data analyses and wrote the manuscript. All authors reviewed and approved this manuscript.

## Conflict of Interest Statement

The authors declare that the research was conducted in the absence of any commercial or financial relationships that could be construed as a potential conflict of interest.

## Footnotes

1 http://www.oie.int/fileadmin/Home/eng/Health_standards/tahm/2.03.04_AI.pdf

## References

[B1] Abdelwhab E. M. Veits J. Mettenleiter T. C. (2014). Prevalence and control of H7 avian influenza viruses in birds and humans. *Epidemiol. Infect.* 142 896–920. 10.1017/S0950268813003324 24423384 PMC9151109

[B2] Banks J. Speidel E. Alexander D. J. (1998). Characterisation of an avian influenza A virus isolated from a human–is an intermediate host necessary for the emergence of pandemic influenza viruses? *Arch. Virol.* 143 781–787. 10.1007/s007050050329 9638147

[B3] Chen Y. Liang W. Yang S. Wu N. Gao H. Sheng J. (2013). Human infections with the emerging avian influenza A H7N9 virus from wet market poultry: clinical analysis and characterisation of viral genome. *Lancet* 381 1916–1925. 10.1016/S0140-6736(13)60903-4 23623390 PMC7134567

[B4] Graaf A. Beer M. Harder T. (2017). Real-time reverse transcription PCR-based sequencing-independent pathotyping of Eurasian avian influenza A viruses of subtype H7. *Virol. J.* 14:137. 10.1186/s12985-017-0808-3 28738896 PMC5525275

[B5] Harlow E. Lane D. (1988). *Antibodies: A Laboratory Manual*. New York, NY: Cold Spring Harbor Laboratory Press.

[B6] Hoffmann E. Neumann G. Kawaoka Y. Hobom G. Webster R. G. (2000). A DNA transfection system for generation of influenza A virus from eight plasmids. *Proc. Natl. Acad. Sci. U.S.A.* 97 6108–6113. 10.1073/pnas.100133697 10801978 PMC18566

[B7] Hoffmann E. Stech J. Guan Y. Webster R. G. Perez D. R. (2001). Universal primer set for the full-length amplification of all influenza A viruses. *Arch. Virol.* 146 2275–2289. 10.1007/s007050170002 11811679

[B8] Influenza A. (2012). *OIE Manual of Diagnostics Tests and Vaccines for Terrestrial Animals*, 6th Edn. Paris: OIE, 465–481.

[B9] Jia W. Cao C. Lin Y. Zhong L. Xie S. Wang X. (2017). Detection of a novel highly pathogenic H7 influenza virus by duplex real-time reverse transcription polymerase chain reaction. *J. Virol. Methods* 246 100–103. 10.1016/j.jviromet.2017.03.014 28411129

[B10] Jin C. Wu N. Peng X. Yao H. Lu X. Chen Y. (2014). Comparison of a new gold immunochromatographic assay for the rapid diagnosis of the novel influenza A (H7N9) virus with cell culture and a real-time reverse-transcription PCR assay. *Biomed. Res. Int.* 2014:425051. 10.1155/2014/425051 24822207 PMC4009114

[B11] Ju Y. Hao H. J. Xiong G. H. Geng H. R. Zheng Y. L. Wang J. (2010). Development of colloidal gold-based immunochromatographic assay for rapid detection of *Streptococcus suis* serotype 2. *Vet. Immunol. Immunopathol.* 133 207–211. 10.1016/j.vetimm.2009.08.010 19733402

[B12] Kang K. Chen L. Zhao X. Qin C. Zhan Z. Wang J. (2014). Development of rapid immunochromatographic test for hemagglutinin antigen of H7 subtype in patients infected with novel avian influenza A (H7N9) virus. *PLoS One* 9:e92306. 10.1371/journal.pone.0092306 24647358 PMC3960227

[B13] Kurtz J. Manvell R. J. Banks J. (1996). Avian influenza virus isolated from a woman with conjunctivitis. *Lancet* 348 901–902. 10.1016/S0140-6736(05)64783-6 8826845

[B14] Liu D. Zhang Z. He L. Gao Z. Li J. Gu M. (2018). Characteristics of the emerging chicken-origin highly pathogenic H7N9 viruses: a new threat to public health and poultry industry. *J. Infect.* 76 217–220. 10.1016/j.jinf.2017.09.005 28941628

[B15] Liu J. Yao L. Zhai F. Chen Y. Lei J. Bi Z. (2018). Development and application of a triplex real-time PCR assay for the simultaneous detection of avian influenza virus subtype H5, H7 and H9. *J. Virol. Methods* 252 49–56. 10.1016/j.jviromet.2017.11.005 29129489

[B16] Liu M. Song T. Hua S. Wu A. Jiang T. (2015). Computational analysis of antigenic epitopes of avian influenza A (H7N9) viruses. *Sci. China Life Sci.* 58 687–693. 10.1007/s11427-015-4886-4 26100010

[B17] Manzoor R. Sakoda Y. Sakabe S. Mochizuki T. Namba Y. Tsuda Y. (2008). Development of a pen-site test kit for the rapid diagnosis of H7 highly pathogenic avian influenza. *J. Vet. Med. Sci.* 70 557–562. 10.1292/jvms.70.557 18628595

[B18] Matrosovich M. N. Gambaryan A. S. Teneberg S. Piskarev V. E. Yamnikova S. S. Lvov D. K. (1997). Avian influenza A viruses differ from human viruses by recognition of sialyloligosaccharides and gangliosides and by a higher conservation of the HA receptor-binding site. *Virology* 233 224–234. 10.1006/viro.1997.8580 9201232

[B19] Munch M. Nielsen L. P. Handberg K. J. Jorgensen P. H. (2001). Detection and subtyping (H5 and H7) of avian type A influenza virus by reverse transcription-PCR and PCR-ELISA. *Arch. Virol.* 146 87–97. 10.1007/s007050170193 11266220

[B20] Nakajima S. Nakajima K. Nobusawa E. Zhao J. Tanaka S. Fukuzawa K. (2007). Comparison of epitope structures of H3HAs through protein modeling of influenza A virus hemagglutinin: mechanism for selection of antigenic variants in the presence of a monoclonal antibody. *Microbiol. Immunol.* 51 1179–1187. 10.1111/j.1348-0421.2007.tb04013.x 18094536

[B21] Paek S. H. Lee S. H. Cho J. H. Kim Y. S. (2000). Development of rapid one-step immunochromatographic assay. *Methods* 22 53–60. 10.1006/meth.2000.1036 11020318

[B22] Quan C. Shi W. Yang Y. Yang Y. Liu X. Xu W. (2018). New threats of H7N9 influenza virus: the spread and evolution of highly and low pathogenic variants with high genomic diversity in Wave Five. *J. Virol.* 92 e301–e318. 10.1128/JVI.00301-18 29563296 PMC5952148

[B23] Reed L. J. Muench H. (1938). A simple method of estimating fifty percent endpoints. *Am. J. Hyg.* 27 493–496.

[B24] Shi J. Z. Deng G. H. Liu P. H. Zhou J. P. Guan L. Z. Li W. H. (2013). Isolation and characterization of H7N9 viruses from live poultry markets-implication of the source of current H7N9 infection in humans. *Chin. Sci. Bull.* 58 1857–1863. 10.1007/s11434-013-5873-4

[B25] Shien J. H. Fu L. F. Wu J. R. Cheng M. C. Shieh H. K. Chang P. C. (2008). Development of blocking ELISA for detection of antibodies against avian influenza virus of the H7 subtype. *J. Microbiol. Immunol. Infect.* 41 369–376. 19122917

[B26] Throsby M. van den Brink E. Jongeneelen M. Poon L. L. Alard P. Cornelissen L. (2008). Heterosubtypic neutralizing monoclonal antibodies cross-protective against H5N1 and H1N1 recovered from human IgM+ memory B cells. *PLoS One* 3:e3942. 10.1371/journal.pone.0003942 19079604 PMC2596486

[B27] Tsuchiya E. Sugawara K. Hongo S. Matsuzaki Y. Muraki Y. Li Z. N. (2001). Antigenic structure of the haemagglutinin of human influenza A/H2N2 virus. *J. Gen. Virol.* 82(Pt 10), 2475–2484. 10.1099/0022-1317-82-10-2475 11562540

[B28] Velumani S. Du Q. Fenner B. J. Prabakaran M. Wee L. C. Nuo L. Y. (2008). Development of an antigen-capture ELISA for detection of H7 subtype avian influenza from experimentally infected chickens. *J. Virol. Methods* 147 219–225. 10.1016/j.jviromet.2007.09.004 17950911

[B29] Wang X. Jiang H. Wu P. Uyeki T. M. Feng L. Lai S. (2017). Epidemiology of avian influenza A H7N9 virus in human beings across five epidemics in mainland China, 2013-17: an epidemiological study of laboratory-confirmed case series. *Lancet Infect. Dis.* 17 822–832. 10.1016/S1473-3099(17)30323-7 28583578 PMC5988584

[B30] Zhang F. Bi Y. Wang J. Wong G. Shi W. Hu F. (2017). Human infections with recently-emerging highly pathogenic H7N9 avian influenza virus in China. *J. Infect.* 75 71–75. 10.1016/j.jinf.2017.04.001 28390707

