# Supplementary material for: Development of a Colloidal Gold-Based Immunochromatographic Strip for Rapid Detection of H7N9 Influenza Viruses
Source: Front Microbiol. 2018 Aug 31;9:2069. doi: 10.3389/fmicb.2018.02069 (PMC6127252; doi:10.3389/fmicb.2018.02069)
Supplement: Supplementary file 1 [file Table_1.DOCX]

**Development of a Colloidal Gold-Based Immunochromatographic Strip for Rapid Detection of H7N9 influenza viruses**

Zhihao Sun^1，2，3^, Baolan Shi^5^, Feifei Meng^1，2，3^, Ruonan Ma^1，2，3^, Qingyun Hu^1，2，3^, Tao Qin^1，2，3, 4^, Sujuan Chen^1，2，3，4^, Daxin Peng^1，2，3，4^, Xiufan Liu^1，2，3，4^

**Supplementary Table 1.** The strains and titers of the avian influenza viruses used in this study

| Viruses | Subtype | HA titer (log2) |
| --- | --- | --- |
| A/Duck/Eastern China/103/2003 | H1N1 | 7 |
| A/Duck/Eastern China/866/2003 | H3N2 | 6 |
| A/Duck/Eastern China/160/2002 | H4N6 | 5 |
| A/Duck/Eastern China/59/2005 | H5N1 | 7 |
| A/Duck/Eastern China/164/2002 | H6N2 | 7 |
| A/Duck/Eastern China/01/2005 | H8N4 | 6 |
| A/Duck/Eastern China/01/2000 | H9N2 | 9 |
| A/Duck/Eastern China/488/2003 | H10N3 | 6 |
| A/Duck/Eastern China/05/2005 | H11N2 | 6 |
| A/Duck/Eastern China/37/2015 | H12N5 | 7 |
| A/Chicken/Jiangsu/JT/2013 | H7N9 | 8 |
| A/Chicken/Jiangsu/DT/2013 | H7N9 | 8 |
| A/Chicken/Jiangsu/WX/2013 | H7N9 | 7 |
| A/Chicken/Jiangsu/TM/2013 | H7N9 | 8 |
| A/Chicken/Jiangsu/WJ-1/2013 | H7N9 | 6 |
| A/Chicken/Jiangsu/WJ-2/2013 | H7N9 | 8 |
| A/Chicken/Jiangsu/TC/2013 | H7N9 | 8 |
| A/Chicken/Jiangsu/XZ-1/2013 | H7N9 | 7 |
| A/Chicken/Jiangsu/XZ-2/2013 | H7N9 | 7 |
| A/Chicken/Jiangsu/JX05/2014 | H7N9 | 7 |
| A/Chicken/Zhejiang/ZJ03/2014 | H7N9 | 6 |
| A/Chicken/Jiangsu/JY18/2014 | H7N9 | 8 |
| A/Chicken/Jiangsu/JY10/2014 | H7N9 | 7 |
| A/Chicken/Jiangsu/NT22/2014 | H7N9 | 7 |
| A/Chicken/Jiangsu/CZ04/2014 | H7N9 | 6 |
| A/Chicken/Jiangsu/XZ19/2014 | H7N9 | 7 |
| A/Chicken/Shandong/SD20/2014 | H7N9 | 7 |
| A/Chicken/Jiangsu/W1-8/2015 | H7N9 | 8 |
| A/Chicken/Jiangsu/WJ14/2015 | H7N9 | 7 |
| A/Chicken/Jiangsu/JY16/2016 | H7N9 | 6 |
| A/Chicken/Jiangsu/WX11/2016 | H7N9 | 7 |
| A/Chicken/Jiangsu/MC14/2017 | H7N9 | 7 |
| A/chicken/Hebei/XT-3/2017 | H7N9 | 5 |
